# Self-similarity study based on the particle sizes of coal-series diatomite

**DOI:** 10.1038/s41598-024-57710-x

**Published:** 2024-03-29

**Authors:** Liang Cheng, Guangming Wang, Zhijun Ma, Hao Guo, Ye Gao, Qi Zhang, Jing Gao, Hanghang Fu

**Affiliations:** 1 College of Mining, Liaoning Technical University, Fuxin, 123000 China; 2 College of Material Science and Engineering, Liaoning Technical University, Fuxin, 123000 China; 3 School of Resources and Civil Engineering, Northeastern University, Shenyang, 110003 China; 4 Liaoning Institute of Geology and Mineral Resources Co., Ltd, Shenyang, 110032 China

**Keywords:** Environmental sciences, Mineralogy

## Abstract

Coal-series diatomite (CSD) is widely distributed in China and has poor functional and structural properties and exhibits limited utilization of high value-added materials, resulting in a serious waste of resources and tremendous pressure on the environment. Moreover, due to differences in the mineralogical characteristics of CSD, different particle size scales (PSSs) have different functional structures and exhibit different self-similarities. In this study, we took CSD as the research object and PSS as the entry point and carried out a self-similarity study based on gas adsorption and an image processing method to illustrate the microstructures and self-similarities of different PSSs. The results showed that the pore structure of the CSD was dominated by mesopores and macropores and basically lacked micropores. The fractal dimensions were calculated with the Frenkel-Haisey-Hill (FHH) model and Menger model, and the *D*_F1_ values for − 0.025 mm and − 2 mm were 2.51 and 2.48, respectively, and the *D*_M1_ values were 3.75 and 3.79, respectively, indicating that the mesopore structure of the fine PSS was complex, whereas macropores were present in the coarse PSS. MATLAB was programmed to obtain grayscale thresholds, binarized images, grayscale histograms, three-dimensional (3D) reconstruction images and box dimensions, which enabled us to observe the microstructures and self-similarities of the CSD. Self-similarity studies based on particle sizes are very important for functional application of CSD.Please note that article title mismatch between MS and JS we have followed MS, kindly check and cofirm.Yes, I have checked and confirmed.Kindly check and confirm corresponding author mail id are correctly identified.Yes, I have checked and confirmed.

The study of self-similarity, i.e., local and global similarity, allows quantitative characterization of surface roughnesses and the inhomogeneity of pore structures, reflecting the validity and irregularity of the spaces occupied by complex forms^1–3^. CSD is a porous mineral widely distributed in China, with complex associative relationships, poor functional structure, and low utilization of high value-added materials, which is very likely to cause a serious waste of resources and tremendous pressure on the environment if it is disposed of as a coal-series waste through open piling and backfilling of mining areas^4,5^. Use of the primary minerals in diatomite causes problems such as resource depletion, mining difficulty, and environmental pollution during development and utilization, while the low cost of mining CSD compensates for resource depletion and environmental damage and enables resourceful utilization of coal-series wastes^6–8^. Diatomite is a natural porous mineral with a unique 3D pore structure, and it exhibits properties such as high porosity, large *SSA*, and chemical stability , and it has been widely used in filter aids^9^, adsorbents^10^, carriers^11^, and porous ceramics^12^. Diatomite is an important mineral resource and functional mineral material for modern industry^13–16^. The characteristic parameters of the pore structure are mainly the *SSA*, pore volume, average pore diameter, most probable diameter, pore diameter distribution and surface roughness^17–20^. The technology of diatomite-based porous mineral materials is developing rapidly, but the primary mineral resources of high-quality diatomite are becoming scarce, and there is an urgent need to find CSD self-similar functional structures and utilize theory and evaluation indices and to solve the resource bottleneck problem.

The development and utilization of a self-similarity functional structure for CSD generally includes 2 stages: purification of the raw soil and preparation of the materials. (1) Raw soil purification refers to the selection of suitable technology to separate associated minerals, such as metal minerals, clay minerals, detrital minerals, and organic matter. Purification increases the contents of useful minerals such as opal and others in the diatomite, separates consociated gangue minerals, and improves the 3D pore structures of clogs and encapsulants. Opal is the main constituent mineral of the diatom skeleton, and its chemical composition is SiO_2_-nH_2_O; the SiO_2_ content can be used to indirectly evaluate the self-similarity pore structure of diatomite. Sun et al.^21^ found that the effect of feed concentration on the SiO_2_ content of the concentrate during centrifugal purification of diatomite was most significant by using response surface methodology, and the content increased from 80.17% to 86.89% with a significant increase in porosity. Chen et al.^22^ used hydraulic dispersion, high-temperature calcination, and ultrasonic cavitation to purify clayey diatomite; the SiO_2_ content increased from 63.54% to 90.95%, the *SSA* increased from 66.65 m^2^·g^−1^ to 111.53 m^2^·g^−1^, and a good pore diameter distribution was obtained. Wakeel^23^ used centrifugation and calcination to increase the SiO_2_ content of Nile diatomite from 73.64% to 87.60%, the *SSA* from 55.66 m^2^·g^−1^ to 74.18 m^2^·g^−1^, and the pore volume from 0.0176 cm^3^·g^−1^ to 0.023 cm^3^·g^−1^ at low cost to obtain products that met the need for self-similar functional structures in different industries. (2) Material preparation refers to use of the unique 3D pore structure of diatomite to load active sites and structures onto diatomite carriers. Due to their self-similarity functional structure, diatomite-based materials exhibit excellent synergistic effects and diverse uses. Dispersion and agglomeration of diatomite-based material particles are the key factors affecting the performance, and the self-similarity pore structure of diatomite can be evaluated indirectly with the material properties. Li et al.^24^ synthesized diatomite-based Pb(II)-imprinted materials via surface ion imprinting, and grafting of active components onto the surface of diatomite with self-similarity functional structures to form a uniform distribution of imprinted active sites prevented self-adsorption. Xu et al.^25^ prepared CuFe_2_O_4_/diatomite composites for pesticide removal and reported that a uniform distribution of CuFe_2_O_4_ on the self-similarity functional structure of diatomite mitigated the agglomeration of CuFe_2_O_4_, and the *SSA* increased by 3.8 times (57.20 m^2^·g^−1^); the material exhibited excellent mixed pesticide removal and interference resistance. Ren et al.^26^ prepared diatomite-based materials by hydrothermal modification of CaO; the rate of methylene blue adsorption was increased by nearly 7 times, and the formation of calcium alumina silicate and hard calcium silicate on the surface of the self-similarity material increased the *SSA* and pore volume.

Microstructural research methods are mainly qualitative or quantitative studies of microstructural features and their evolution, while macroscopic properties are manifestations of the microstructures^27^. In essence, raw soil purification and material preparation of diatomite mainly involve the development and use of the 3D pore structures, in which the differential 3D pore structures have different self-similarity functional uses. Therefore, pore structure parameters and fractal dimensions are of great significance for the development and utilization of diatomite. Hu et al.^28^ analysed the pore structure of diatomite via N_2_ adsorption and desorption and established a relationship between the pore structure and moisture regulation. Jiang et al.^29^ reported that the pore diameter distribution affected the self-similarity pore structures of diatomite-based porous ceramics. This directly determined the application areas of the porous ceramics, in which the CO_2_ gas released by CaCO_3_ affected the self-similarity porosity (61.61–67.53%) and the pore diameter distribution (1.2~26.6 μm). Smith et al.^30^ analysed the self-similarity fractal dimensions of traditional triblock copolymer mesoporous silicates based on the FHH model for N_2_ adsorption–desorption. Sun et al.^31^ used the Menger model to calculate the fractal dimensions of complex gelling systems and establish the relationship between the self-similarity fractal dimension and the strength, porosity, and *SSA* of the system. Wang et al.^32^ analysed the self-similarity fractal characteristics and pore structures of natural sedimentary diatomites under different consolidation pressures with SEM images, an image segmentation method and an optimal thresholding method and explored the relationship between the fractal box dimension of diatomite and the isotropic consolidation pressure. Niu et al.^33^ and Zhang et al.^34^ realized 3D visualization of the microstructures of haematite flocs and soft soils, respectively, based on SEM images. 3D visualization of the microstructure can reveal self-similarity functional structures^35^. Fractal theory can characterize the multidimensional pore structures of porous materials, which studies of the self-similar functional structure of CSD^36^.

Due to differences in the mineralogical characteristics of CSDs, different PSSs have different functional structures and exhibit different self-similarities, which is highly important for solid waste resource utilization. Moreover, traditional research on CSD has been focused on changes in the crystal structures, morphologies, component contents, physicochemical properties, performance, and related mechanisms. However, there have been no reports in the literature on quantitative characterization and self-similarity studies of the functional structure of CSD based on particle size characteristics. In this study, CSD was taken as the research object, and PSS as the entry point, based on gas adsorption methods (Brunauer‒Emmett‒Teller (BET) model, Barrett‒Joyner‒Halenda (BJH) model, FHH model, and Menger model), an image segmentation method, an optimal thresholding method, a 3D reconstruction method, etc., to illustrate the microstructures and self-similarities of CSDs with different PSSs and to provide fractal geometric evaluation indices for functional applications.

## Experiments and methodology

### Preparation of samples with different PSSs

**Figure 1:**
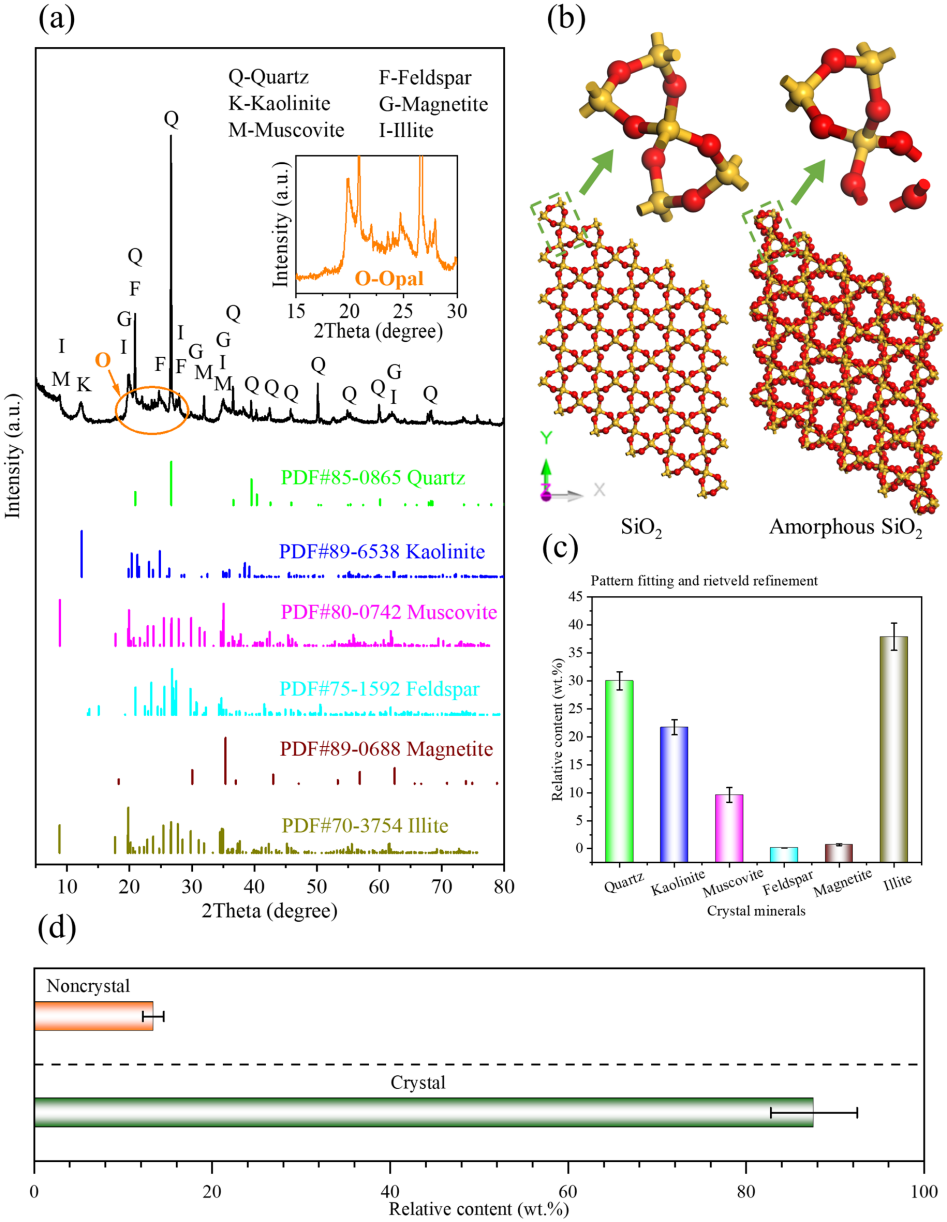
XRD patterns of CSDs (**a**), SiO_2_ crystal (P3121, a=b=4.913 Å, c=5.404 Å^57^) and amorphous structure (**b**), relative content (wt. %) of different crystal minerals based on pattern fitting and Rietveld refinements (**c**), and relative contents (wt. %) of crystalline minerals and noncrystalline minerals (**d**).

The CSD was taken from the Yong'an mining area in Heilongjiang Province, the particle sizes were less than 2 mm, and the diatom particles were incomplete. The chemical composition is shown in Table 1, and the main component was SiO_2_ (59.22%), which also contained impurities such as Al_2_O_3_, Fe_2_O_3_, K_2_O, Na_2_O, CaO, MgO, etc. Meanwhile, the loss on ignition (LOI) was 10.50%. The mineral composition is shown in Fig. 1, in which the main components were opal and quartz, followed by feldspar, mica, montmorillonite, haematite, magnetite and other minerals, and the content of opal was approximately 12.39%, which accounted for approximately one-eighth of the total mineral content.

**Table 1:** Contents (wt.%) of the major chemical and mineral components (by pattern fitting and Rietveld refinement) of the CSD.

Chemical component	Content (wt.%)	Mineral component	Content (wt.%)
SiO_2_	59.22	Quartz	30.0
Al_2_O_3_	18.32	Kaolinite	21.7
Fe_2_O_3_	6.85	Muscovite	9.6
K_2_O	1.46	Feldspar	0.1
Na_2_O	0.53	Magnetite	0.7
CaO	1.75	Illite	37.9
MgO	0.24	Total	100.00
Total	88.37		
Loss on ignition	10.50

**Figure 2:**
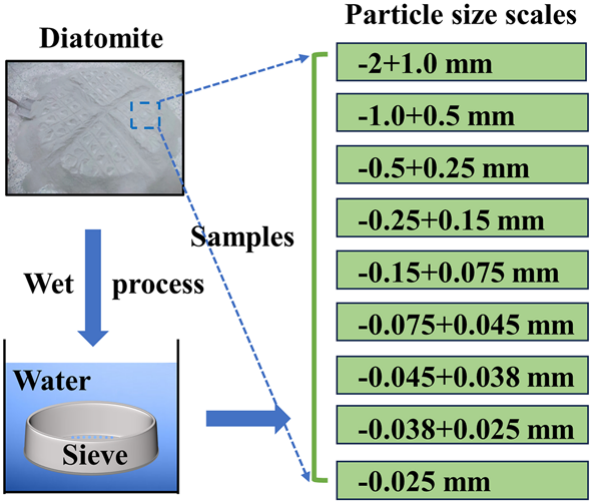
Preparation process of the CSD samples (− 2+1.0 mm, − 1.0+0.5 mm, − 0.5+0.25 mm, − 0.25+0.15 mm, − 0.15+0.075 mm, − 0.075+0.045 mm, − 0.045+0.038 mm, − 0.038+0.025 mm, − 0.025 mm).

The CSD was divided into 9 PSSs, namely, − 2+1 mm, − 1+0.5 mm, − 0.5+0.25 mm, − 0.25+0.15 mm, − 0.15+0.075 mm, − 0.075+0.045 mm, − 0.045+0.038 mm, − 0.038+0.025 mm, and − 0.025 mm, through a set of standard sieves, where "-" indicates a less than relationship and "+" indicates a greater than relationship (Fig. 2).

### Characterization methodology

Based on previous qualitative analyses of CSD, the chemical compositions of the samples were quantitatively analysed with volumetric and gravimetric methods^37^; the mineral compositions and crystal structures of the CSDs were analysed with a D8 ADVANCE X-ray diffractometer (XRD, Bruker, Germany)^38^; and the *SSAs* and pore structures of CSDs with different PSSs were measured with a 3H-2000PMV autospecific surface area analyser (Beishide Instrument S&T. (Beijing) Co., Ltd., China)^39^. First, the total pore volumes (*V*_T_) of the CSD samples were calculated with the BET model based on low-temperature N_2_ isothermal adsorption and desorption data. Second, the average pore diameters (*D*_a_) were calculated based on the *V*_T_ and *SSA*. Finally, the distributions of the *SSAs* and pore diameters for CSDs with different PSSs were analysed with the BJH model^40,41^.

Based on the data from the desorption branches of the N_2_ isothermal adsorption–desorption curves, the FHH model^42–44^ suggested that the gas adsorbed on the fractal surface can be represented as Eq. (1).

1 $${\text{ln}}\left( {\frac{{\text{V}}}{{{\text{V}}_{{0}} }}} \right){\text{ = Aln}}\left[ {{\text{ln}}\left( {\frac{{{\text{p}}_{{0}} }}{{\text{p}}}} \right)} \right]{\text{ + constant }}$$

where *p* is the equilibrium pressure, *V* is the volume of adsorbed N_2_ at the equilibrium pressure, *V*_0_ is the volume of adsorbed N_2_ in the monolayer, *p*_0_ is the saturated N_2_ pressure, ln*V* is linearly fitted to ln[ln(*p*_0_/*p*)], and *A* is a constant related to the fractal dimension (*D’*) and the adsorption mechanism. When the adsorption mechanism is capillary condensation, *D’* can be expressed as in Eq. (2).

2 $${\text{D}}^{\prime } {\text{ = A + 3 }}$$

The pore structure of the CSD was analysed with the Menger model^45^, and Eq. (3) was obtained based on the pore volume V_*p*_(*r*) = *R*^*3*^ - *V*(*r*).

3 $$\lg \left( { - \frac{{{\text{dV}}}}{{{\text{dr}}}}} \right) \sim \left( {2 - {\text{D}}^{\prime } } \right)\lg {\text{r}}$$

Combining the Warshburn equation and Eq. (3) led to Eq. (4).

4 $$\lg \left( {\frac{{{\text{dV}}_{{\text{p}}} }}{{{\text{dp}}}}} \right) \sim \left( {{\text{D}}^{\prime } - 4} \right)\lg {\text{p}}$$

The fractal dimension of a self-similarity pore structure was translated into a measure of the N_2_ pressure and the pore volume, which were derived directly from the slope of a double logarithmic plot of d*Vp*/d*p* vs. *p*^31^.

A Sigma 300 scanning electron microscope (SEM, Zeiss, Germany) was used to measure the pore structures and analyse the self-similarity fractal characteristics of the CSDs with different PSSs^32^. An image that had been stored within the computer, which was composed of pixel points of size k*, was considered. Since the SEM image contained parametric information that could be mistaken for particles and pores during image processing, thus affecting the accuracy of the image, it was necessary to crop the image, subtract the boundaries and leave an accurate image that contained only the CSD image without text, and the image pixels for this experiment were 2837 × 2121. The image was binarized so that each pixel point was either black or white. For this study, the image in jpg format was converted to a grayscale image with the rgb2gray function in MATLAB software. It was first binarized with the optimal segmentation thresholding method to obtain a binarized image with background pixels as particles (white) and target pixels as pores (black). The optimal threshold segmentation method^46,47^ originated from fractal theory in image processing, and the basic principle was to determine a grayscale threshold. Then, the relationship between the size of the pixel grayscale value and the threshold value in the image was determined for image segmentation. Using this method, the boundary contour of the substance was clearly defined, and the image was converted into black and white so that the information of the image was stored in a matrix, with each point representing a pixel of the image.

In binarization of the images, some of the particles were mistaken for apertures when the threshold value was too large; thus, the optimal threshold value was determined to ensure that most of the particles were defined in terms of their contours. The following binarization algorithm with an optimal segmentation threshold was used:

- (1) The minimum and maximum grayscale values *f*_min_ and *f*_max_ in the image were found and denoted the initial value of the threshold, as in Eq. (5).

5 $${\text{l}}_{{1}} { = }\frac{{{\text{f}}_{{{\text{min}}}} + {\text{f}}_{{{\text{max}}}} }}{{2}}$$

- (2) The digital image was divided into foreground and background parts with the threshold value derived in the previous step, and the average grayscale values *f*_A_ and *f*_B_ of the two parts were found with Eqs. (6) and (7).
  6 $${\text{f}}_{{\text{A}}} = \frac{{\mathop \sum \nolimits_{{{\text{f}}({\text{i}},{\text{j}}) < {\text{l}}_{{\text{n}}} }} {\text{f}}({\text{i}},{\text{j}}) \times {\text{w}}({\text{i}},{\text{j}})}}{{\mathop \sum \nolimits_{{{\text{f}}({\text{i}},{\text{j}}) < {\text{l}}_{{\text{n}}} }} {\text{w}}({\text{i}},{\text{j}})}}$$
  7 $${\text{f}}_{{\text{B}}} = \frac{{\sum\nolimits_{{{\text{f}}({\text{i}},{\text{j}}) > {\text{l}}_{{\text{n}}} }} {\text{f}} ({\text{i}},{\text{j}}) \times {\text{w}}({\text{i}},{\text{j}})}}{{\sum\nolimits_{{{\text{f}}({\text{i}},{\text{j}}) > {\text{l}}_{{\text{n}}} }} {\text{w}} ({\text{i}},{\text{j}})}}$$
  where *f*(*i*,*j*) is the grayscale value of the (*i*,*j*) point corresponding to the planar image and *w*(*i*,*j*) is the corresponding weight coefficient, which is generally taken as *w*(*i*,*j*)=1.
- (3) The new threshold was found; $l_{{n + 1}} = (f_{A} {\text{ }} + {\text{ }}f_{B} )/2;{\text{if}}|l_{{n + 1}} - ln|{\text{ }} \le {\text{ }}0.001$, then the loop ended; otherwise, step (2) was used for recalculation.

The SEM image was first converted into a grayscale image, in which the darker grayscale region was the pores, and the grayscale value was analysed. Then, the threshold *l* was obtained with the above algorithm and Eq. (8) to extract the microstructural information in the SEM image.

8 $${\text{ f(i,j) = }}\left\{ {\begin{array}{*{20}c} {{\text{0 f(i,j) }} \le {\text{ l}}} \\ {\text{1 f(i,j) > l}} \\ \end{array} } \right.$$

where *l* is the threshold value obtained with the optimal segmentation thresholding algorithm. A program in MATLAB was used to convert the above binarized image into a data file, in which the number of rows and columns corresponded to the binarized image, and each frame of data was assigned a value of 1 or 0 depending on the colour of the pixel point to which it corresponded (Fig. 6); 1 indicated that the corresponding pixel point in the binarized image was white and 0 indicated that it was black.

Self-similarity fractal features are objective characteristics of many natural things and phenomena, and physical fractals that exist in nature tend to show scale and randomness; i.e., they only show fractal features statistically at a particular scale. Characterisation of the fractal for a research object depends on the characteristics of the object as well as the purpose of the research, and different descriptive methods can be used to compute the fractal dimensions, such as the Hausdorff dimension^48^ and similarity dimension^49^. However, for many fractals, both of these dimensions are difficult to compute; in practice, the box dimension^32,50^ is generally used, and one of its equivalent formulas is shown in Eq. (9).

9 $$\lg {\text{N}}_{{\text{k}}} = {\text{C}} - {\text{D}}^{*} \lg {\text{k}}$$

where *C* is a constant; *k* is the size of the box; and *N*_*k*_ indicates the number of boxes of size *k* needed to cover all foreground images. In the calculation, the data file is divided into blocks, each of which is a square with side lengths containing pixels of number *n*. The number of blocks containing 0 is denoted as *N*_*kn*_. If the size of a pixel point is k*, the length of a block with *n* rows and columns is *k*_*n*_ = nk*. The corresponding number of boxes can be obtained by using *k*_*n*_ as the edge length for block division.

In 3D reconstructions of SEM images with MATLAB software^51–53^, the first step was to collect and process the 3D image data, and then, according to the imaging principle, calculate the action of all pixels on the light to obtain the two-dimensional (2D) projection image and construct the 3D body reconstruction fragments. The 3D display effect was set up to complete the 3D reconstruction of SEM images. The body projection method was mostly used to calculate the projection of an object on a plane^54,55^. By projecting points on the surface of the 3D object, i.e., the 3D data points after preprocessing, along the parallel lines to the 2D plane, and by transforming the angle between the parallel lines and the 3D object so that all of the 3D data points were projected on the 2D surface, the projection principle is shown in Fig. 13b. The conversion from a 2D to 3D display was realized by continuously projecting the map. The operation was simplified by the MATLAB software, and the projection calculation was completed directly through the isosurface function, a function tool for drawing 3D implicit function images.

## Results and discussion

### Pore structure analyses

**Figure 3:**
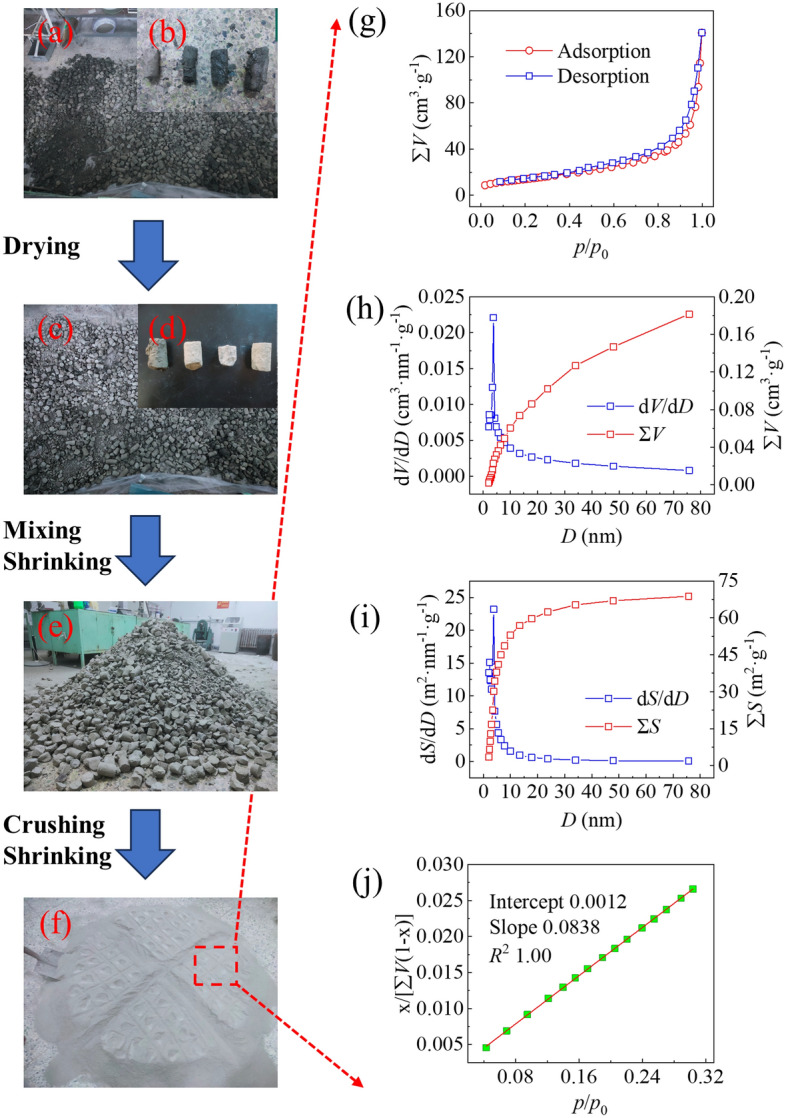
Samples of raw soil (**a**, **b**), samples after drying (**c**, **d**), samples after mixing and shrinking (**e**), samples after crushing and shrinking to − 2 mm particle sizes (**f**), isothermal adsorption desorption curve (**g**), pore diameter distribution curves of the BJH Model (h), SSA distribution curves of the BJH Model (**i**), and SSA fitting curve of the BET Model (**j**).

Fig. 3 shows the results of N_2_ isothermal adsorption and detachment of the CSD after drying, mixing, condensation, and crushing to particle sizes of less than 2 mm. As shown in Fig. 3a-f, the CSD had different colours, such as black‒grey, dark-grey, and white‒grey, which was attributed to nonuniformity of the water contents of the samples. As shown in Fig. 3g, the N_2_ isothermal adsorption-desorption isotherms of the CSDs exhibited type IV isothermal curves, and there were obvious hysteresis loops in the relative pressure (*p*/*p*_0_) range 0.6–0.9, indicating the presence of a typical mesoporous structure in the CSD^56^. The isothermal adsorption desorption curves did not show high plateaus at high relative pressures (*p*/*p*_0_ > 0.9), indicating the presence of macropores in the CSD. Little N_2_ was adsorbed at low relative pressures (*p*/*p*_0_ < 0.1), indicating that the microporous structure in the CSD was not developed. The geometric nonuniformity of the pore structures determined that a single pore diameter did not accurately characterize the real pore distribution, and a reasonable model was needed to analyse the pore diameter distribution. Fig. 3h,i shows the pore diameter distribution curves and the *SSA* vs. pore diameter distribution curves of CSDs analysed by the BJH model. As shown in Fig. 3h, there was an obvious peak for d*V*/d*D* in the range of pore diameters from 0 nm to 10 nm, indicating that the pore distribution in this range was more concentrated. As shown in Fig. 3i, the d*S*/d*D* plot of the CSD had an obvious peak in the pore diameter range 2 nm to 5 nm, indicating that the pore distribution in this range was also more concentrated. Moreover, when the pore diameter was larger than 10 nm, a part of the total *SSA* distribution curve for the CSD still existed in the pore diameter range, but the proportion of *SSA* was smaller than that of the *SSA* distribution for the pore diameters in the range 2–10 nm, which suggested that the *SSA* of CSD was mainly determined by the mesopores with smaller diameters. As shown in Fig. 3j, the *SSA* curve showed a good fit with a correlation coefficient (*R*^2^) of 0.9999, a slope (*a*) of 0.0838, an intercept (*b*) of 0.0012, and a BET constant (*c*) of 74.1 in the range of relative pressures 0.04 < *p*/*p*_0_ < 0.32. Using Eq. (10), the *SSA* was calculated as 51.2128 m^2^·g^−1^.

10 $$\frac{{\text{p}}}{{{\text{V}}({\text{p}}_{0} - {\text{p}})}} = \frac{1}{{{\text{V}}_{{\text{m}}} \times {\text{c}}}} + \frac{c - 1}{{{\text{V}}_{{\text{m}}} \times {\text{c}}}} \times \frac{{\text{p}}}{{{\text{p}}_{0} }} \to {\text{V}}_{{\text{m}}} = \frac{1}{{{\text{a}} + {\text{b}}}} \to {\text{ SSA}} = 4.35{\text{V}}_{{\text{m}}}$$

where *p* is the pressure after adsorption equilibrium; *p*_0_ is the saturated vapour pressure of the adsorbent at the adsorption temperature; *V*_m_ is the saturated adsorption capacity for a single layer of the adsorbed material; 4.35 is the area occupied by a single-molecule layer of 1 mL N_2_ under standard conditions; and *V* is the amount of adsorbate on the adsorbent at the adsorption equilibrium pressure.

**Figure 4:**
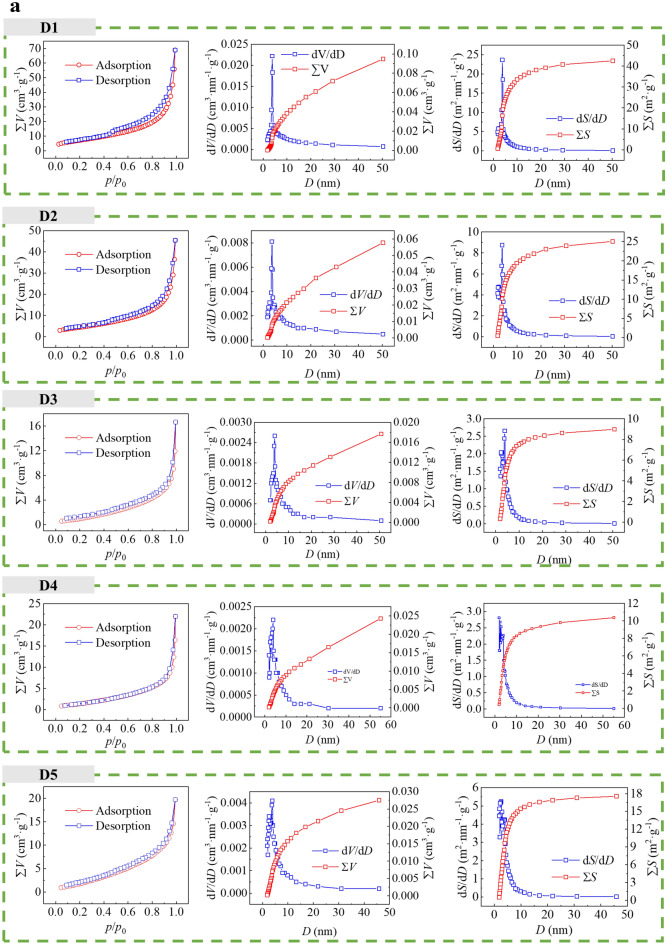

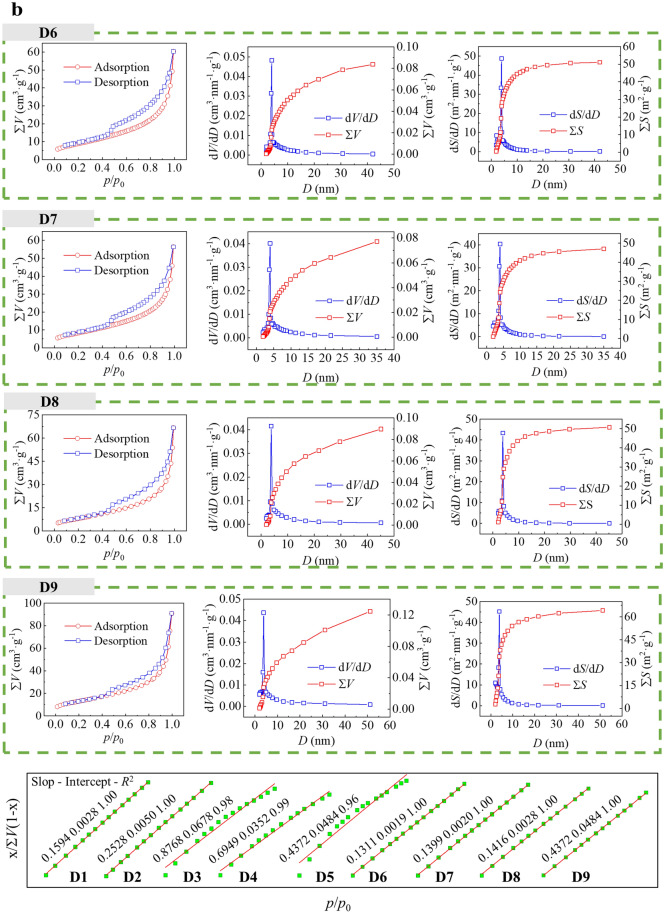
(**a**) Isothermal adsorption-desorption curves, pore diameter distribution curves from the BJH model, and SSA distribution curves from the BJH model for CSDs with different PPSs (D1: − 2+1 mm, D2: − 1+0.5 mm, D3: − 0.5+0.25 mm, D4: − 0.25+0.15 mm, D5: − 0.15+0.075 mm). (**b)** Isothermal adsorption desorption curves, BJH model pore diameter distribution curves, BJH model SSA distribution curves, and SAA fitting curves from the BET model for CSDs with different PPSs (D1: − 2+1 mm, D2: − 1+0.5 mm, D3: − 0.5+0.25 mm, D4: − 0.25+0.15 mm, D5: − 0.15+0.075 mm, D6: − 0.075+0.045 mm, D7: − 0.045+0.038 mm, D8: − 0.038+0.025 mm, D9: − 0.025 mm).

Fig. 4a, b shows the N_2_ isothermal adsorption and desorption data for CSDs with different PPSs. The isothermal adsorption and desorption curves, pore diameters and *SSAs* of the CSDs at 9 PPS reflected similar changes in the pore structures. As the particle sizes of the CSDs decreased, the corresponding hysteresis loop expanded, the pore diameters decreased, and the *SSA* increased. The isothermal adsorption and desorption curves of CSDs with different PPSs were indicated type IV isothermal curves, and were hysteresis loops within the range of relative pressures *p*/*p*_0 =_ 0.6–0.9, which indicated that there was a typical mesoporous structure in the CSD. An obvious hysteresis loop was observed in the isothermal adsorption desorption curve of the − 0.025 mm sample, indicating that the mesopores for the CSD with a fine PPS were obviously more abundant, which was attributed to a reduction in gangue minerals, which weakened the agglomeration of CSD particles and resulted in a richer pore structure. The isothermal adsorption desorption curves did not show high plateaus at high relative pressures (*p*/*p*_0_ > 0.9), suggesting that CSD contained macropores. Little N_2_ was adsorbed at low relative pressures (*p*/*p*_0_ < 0.1), indicating that the microporous structure of the CSD was not developed. Moreover, N_2_ adsorption at relative pressures *p*/*p*_0_ = 0.98 increased from 55.8441 cm^3^·g^−1^ for − 2+1 mm to 75.3813 cm^3^·g^−1^ for − 0.025 mm, and N_2_ adsorption for − 0.025 mm was significantly greater than that for the other PPSs, showing an overall increase with decreasing particle sizes. The d*V*/d*D* and d*S*/d*D* for the CSDs with different PPSs had obvious peaks in the range of pore diameters from 0 nm to 10 nm, indicating that the distribution of pores was more concentrated in this range. Moreover, when the pore diameter was larger than 10 nm, a part of the total *SSA* distribution curve for the CSD was still present in the range of pore diameters at different PPSs, but the proportion of the *SSA* accounted for was smaller than that for the *SSA* distribution for pore diameters pf 2 nm to 10 nm, indicating that the *SSAs* of the CSDs with various PPSs were mainly determined by the mesopores with smaller diameters. In addition, the *R*^2^ values of the *SSA* fitted curves at different PPSs in the range of relative pressures 0.04 < *p*/*p*_0_ < 0.32 were greater than 0.9 (Fig. 4b), indicating that the fits were better, the *SSA* increased from 26.8294 m^2^·g^−1^ for − 2+1 mm to 47.0258 m^2^·g^−1^ for − 0.025 mm, and the *SSA* of − 0.025 mm was significantly larger than those of the other PPSs, showing an overall increase with decreasing particle size.

**Figure 5:**
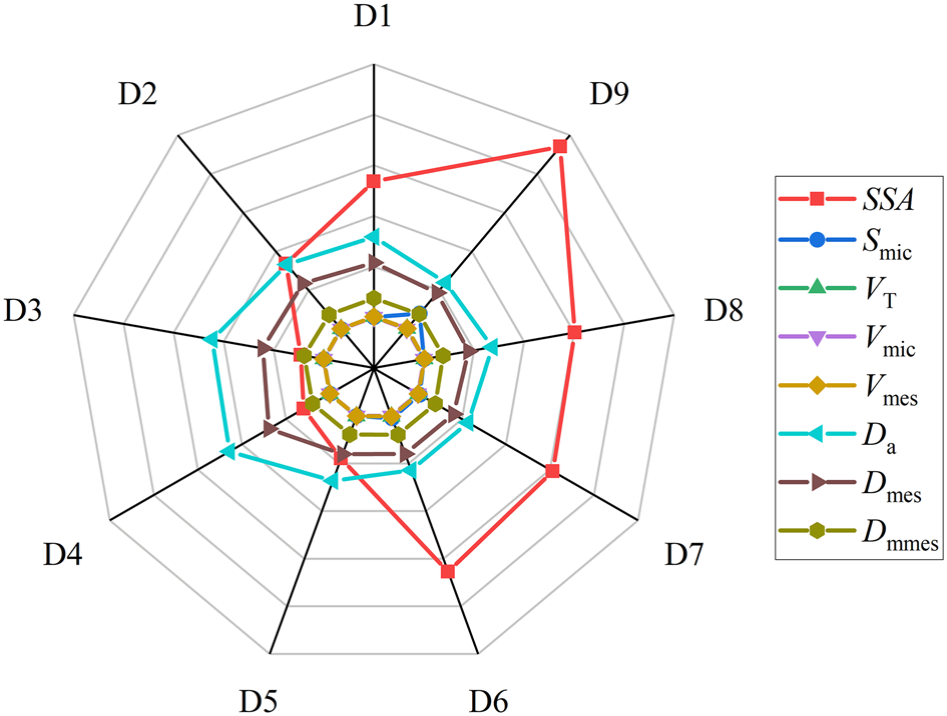
Pore structure parameters of CSD samples with different PSSs determined via low-temperature N_2_ gas adsorption (*SSA* is the specific surface area, *S*_mic_ is the specific surface area of micropores, *V*_T_ is the total pore volume of nitrogen gas adsorption, *V*_mic_ is the microporous pore volume, *V*_mes_ is the mesopore volume, *D*_a_ is the average pore diameter, *D*_mes_ is the mesopore average pore diameter, and *D*_mmes_ is the mesopore most probable diameter). D1: − 2+1 mm, D2: − 1+0.5 mm, D3: − 0.5+0.25 mm, D4: − 0.25+0.15 mm, D5: − 0.15+0.075 mm, D6: − 0.075+0.045 mm, D7: − 0.045+0.038 mm, D8: − 0.038+0.025 mm, D9: − 0.025 mm).

Table 2 and Fig. 5 show the main pore structure parameters of CSDs analysed by the gas adsorption method (N_2_) at different PPSs, in which N_2_ adsorption was positively correlated with the *SSA*, and the *SSA* decreased from 47.0258 m^2^·g^−1^ at − 0.0258 mm to 4.6050 m^2^·g^−1^ at − 0.5+0.25 mm. The hysteresis loops of the isothermal adsorption and desorption isotherms for CSDs with different PPSs in the range of − 0.5 mm generally showed gradual decreases with increasing PSS, indicating that the *V*_mes_ of the CSD basically decreased after grading (Fig. 4a, b). Moreover, the *V*_T_ values of the CSDs with different PSSs in the range of − 0.5 mm decreased with increasing PSS, and the *V*_T_ reached a maximum value of 0.1407 cm^3^·g^−1^ at − 0.025 mm, which was related to the embedded particle sizes of the gangue minerals in the CSDs with different PPSs; as the PSS increased, the pore diameter increased, which increased the pore volume. The t-plot method was used to analyse the microporous structures of CSDs with different PSSs, and the microporous volumes (*V*_mic_) were close to 0 cm^3^·g^−1^, indicating that there were basically no micropores in the CSD. Combined with the N_2_ isothermal adsorption desorption isotherms and pore diameter distribution curves, the pore channels of the CSD were mainly multilevel pore structures composed of mesopores and macropores, and basically, there were no micropores.

**Table 2:** Pore structure parameters of CSDs with different PSSs after low-temperature N_2_ adsorption.

Sample/mm	*SSA*/(m^2^·g^−1^)	*S*_mic_/(m^2^·g^−1^)	
− 0.025	47.0258	4.0315	
− 0.038+0.025	30.1305	0.0000	
− 0.045+0.038	30.6470	0.4057	
− 0.075+0.045	32.7009	0.4778	
− 0.15+0.075	8.9570	0.0000	
− 0.25+0.15	5.9615	0.0000	
− 0.5+0.25	4.6050	0.0000	
− 1+0.5	16.8782	0.0000	
− 2+1	26.8294	0.0000	
− 2	51.2128	0.0000	

Sample/mm	*V*_T_/(cm^3^·g^−1^)	*V*_mic_/(cm^3^·g^−1^)	*V*_mes_/(cm^3^·g^−1^)
− 0.025	0.1407	0.0023	0.1505
− 0.038+0.025	0.1029	0.0000	0.1147
− 0.045+0.038	0.0872	0.0007	0.0962
− 0.075+0.045	0.0934	0.0001	0.1031
− 0.15+0.075	0.0305	0.0000	0.0037
− 0.25+0.15	0.0340	0.0000	0.0370
− 0.5+0.25	0.0257	0.0000	0.0279
− 1+0.5	0.0703	0.0000	0.0752
− 2+1	0.1065	0.0000	0.1161
− 2	0.2177	0.0000	0.2279

Sample/mm	*D*_a_/nm	*D*_mes_/nm	*D*_mmes_/nm
− 0.025	11.9679	9.3405	3.8652
− 0.038+0.025	13.6606	8.9246	3.8417
− 0.045+0.038	11.3812	8.0870	3.9581
− 0.075+0.045	11.4248	7.9886	3.9540
− 0.15+0.075	13.6206	8.0353	3.9009
− 0.25+0.15	22.8131	13.8860	3.9081
− 0.5+0.25	22.3236	12.1837	3.9157
− 1+0.5	16.6605	11.7863	3.7044
− 2+1	15.8781	10.7627	3.7604
− 2	17.0036	13.1792	3.8139

*SSA* is the specific surface area, *S*_mic_ is the specific surface area of the micropores, *V*_T_ is the total pore volume of N_2_ adsorption, *V*_mic_ is the micropore volume, *V*_mes_ is the mesopore volume, *D*_a_ is the average pore diameter, *D*_mes_ is the average mesopore diameter, and *D*_mmes_ is the most probable mesopore diameter.

Please check the layout of tables 2 and 3.Yes, I have checked.

**Figure 6:**
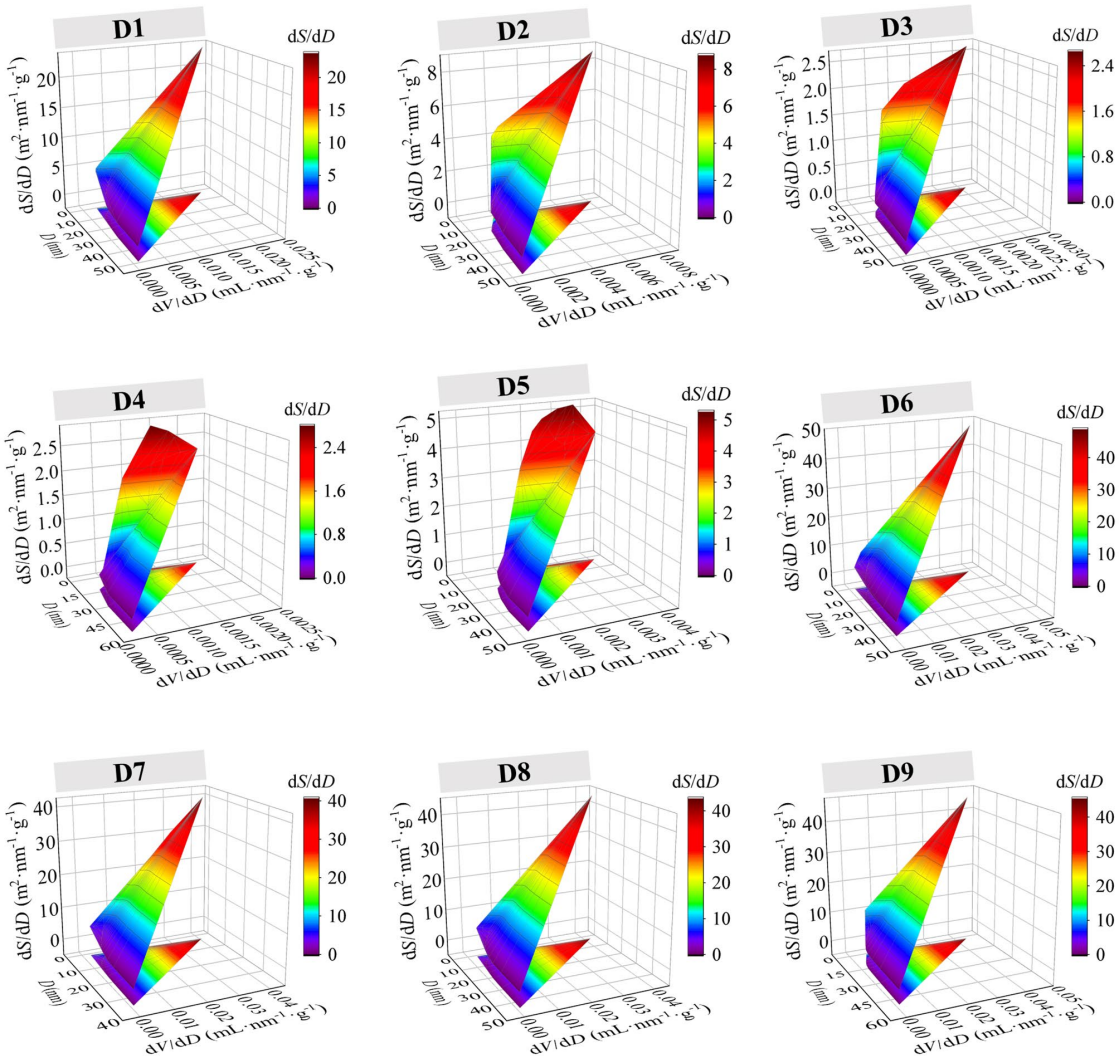
3D spatial surface maps of the pore diameters, volumes and SSAs for CSDs with different PSSs (D1: − 2+1 mm, D2: − 1+0.5 mm, D3: − 0.5+0.25 mm, D4: − 0.25+0.15 mm, D5: − 0.15+0.075 mm, D6: − 0.075+0.045 mm, D7: − 0.045+0.038 mm, D8: − 0.038+0.025 mm, D9: − 0.025 mm).

The formation of the CSD pore structure was attributed to the 3D constitutive units comprising SiO_4_ tetrahedra, which exhibited different pore diameters, pore volumes, and *SSAs* due to plugging and encapsulation of the companion minerals. Fig. 6 shows the 3D spatial surface maps of the pore diameters - pore volumes - *SSAs* for CSDs with different PPSs, which showed that there was a constraint relationship between the pore diameters, pore volumes, and *SSAs*, and smaller pore diameters and larger pore volumes generated larger *SSAs*.

### Fractal characterization with the gas adsorption method

**Figure 7:**
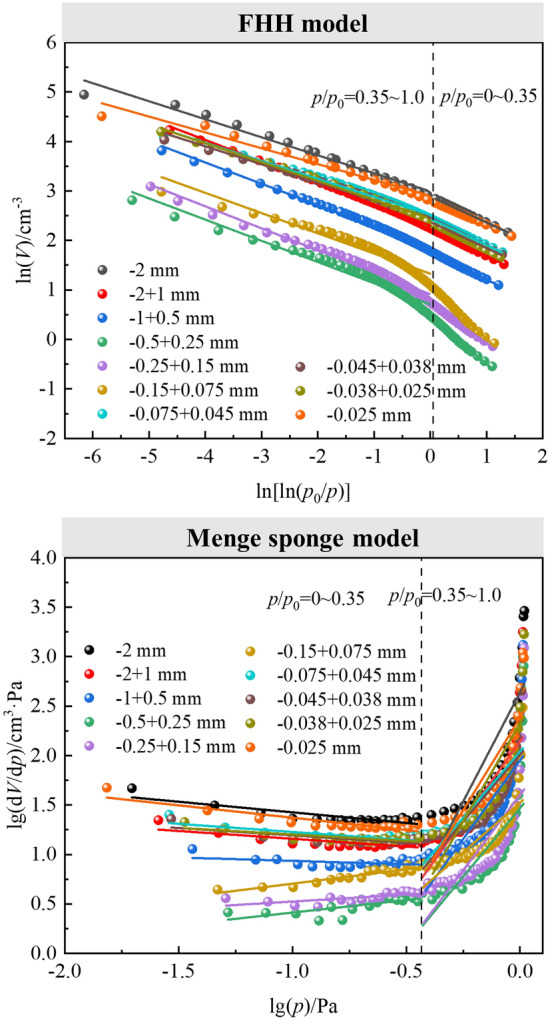
Plots of ln(*V*) vs. ln[ln(*p*_0_/*p*)] (FHH model) and lg(dVp/dp) vs. lg(p) (Menger sponge model) for different PSSs.

There were two distinct linear regions of the isothermal adsorption-desorption isotherms for CSDs with different PPSs, which were located in the relative pressure ranges *p*/*p*_0_ = 0–0.35 and 0.35–1.0 (Fig. 4). With the gas adsorption method, the N_2_ isothermal desorption data for CSDs were different PPSs were fractally fitted with the FHH model, and the FHH fits are shown in Fig. 7. The linear fitting equations, fractal dimensions (*D*_F1_ and *D*_F2_), and fitting correlation coefficients (*R*^2^) are given in Table 3 for the pore channels in CSDs with pore diameters less than 50 nm and different PPSs. The results showed that the CSD had good fits and different fractal characteristics in both regions, with *R*^2^ values greater than 0.93. *D*_F1_ and *D*_F2_ reflect the inhomogeneity of the surface roughnesses and mesopore volumes of the CSD at different PPSs, respectively, and quantitatively characterize the nonhomogeneity and complexity of the pore structure. The fractal dimension influences the interaction of porous mineral surfaces with other substances during physicochemical processes such as adsorption, adhesion and surface diffusion. Table 3 shows the *D*_F1_ and *D*_F2_ values calculated from the fitting results in Fig. 7, with *D*_F1_ = 2.4756 and *D*_F2_ = 2.6358 for − 2 mm and *D*_F1_ = 2.5115 and *D*_F2_ = 2.6799 for − 0.025, in which *D*_-2 mm_ < *D*_-0.025 mm_. The number of mesopores with small pore diameters in the fine PSS was greater, the distribution of the mesopore apertures was narrower, the number of mesopores with large pore diameters in the coarse PSS was greater, and the distribution of mesopore apertures was wider. The increases in D_F1_ with decreasing particle sizes indicated that the surface roughness of the CSD increased with decreasing particle sizes, which was consistent with the change rule for *SSAs* with different PSSs in Table 2 and Fig. 5. In addition, the *D*_F2_ also increased with decreasing particle sizes, and the *D*_F2_ reached the lowest value of 2.6799 at − 0.025 mm.

**Table 3:** Fractal dimension (*D*_F_ = 3 + *A*, *D*_M_ = 4 + *A*) of CSDs with different PSSs derived with the FHH model and Menger model.

Size/mm	*P*/*P*_0_=0–0.35			*P*/*P*_0_=0.35–1.0		
	FHH	*D*_F1_	*R*_1_^2^	FHH	*D*_F2_	*R*_2_^2^
− 2	y_1_=− 0.52x_1_+2.89	2.4756	1.00	y_2_=− 0.36x_2_+2.99	2.6358	0.98
− 2+1	y_1_=− 0.56x_1_+2.24	2.4431	1.00	y_2_=− 0.43x_2_+2.30	2.5669	1.00
− 1+0.5	y_1_=− 0.57x_1_+1.77	2.4350	1.00	y_2_=− 0.43x_2_+1.86	2.5707	0.99
− 0.5+0.25	y_1_=− 0.96x_1_+0.46	2.0440	1.00	y_2_=− 0.43x_2_+0.71	2.5716	0.96
− 0.25+0.15	y_1_=− 0.81x_1_+0.74	2.1857	1.00	y_2_=− 0.44x_2_+0.91	2.5556	0.98
− 0.15+0.075	y_1_=− 1.08x_1_+1.09	1.9159	1.00	y_2_=− 0.41_2_+1.33	2.5915	0.94
− 0.075+0.045	y_1_=− 0.53x_1_+2.44	2.4682	1.00	y_2_=− 0.36x_2_+2.52	2.6355	0.99
− 0.045+0.038	y_1_=− 0.53x_1_+2.37	2.4735	1.00	y_2_=− 0.36x_2_+2.45	2.6371	0.99
− 0.038+0.025	y_1_=− 0.57x_1_+2.35	2.4252	1.00	y_2_=− 0.38x_2_+2.44	2.6199	0.99
− 0.025	y_1_=− 0.49x_1_+2.80	2.5115	1.00	y_2_=− 0.32x_2_+2.90	2.6799	0.97

Size/mm	*p*/*p*_0_=0–0.35			*p*/*p*_0_=0.35–1.0		
	Menger	*D*_M1_	*R*_1_^2^	Menger	*D*_F2_	*R*_2_^2^
− 2	y_1_=− 0.21x_1_+1.21	3.7856	0.74	y_2_=4.42x_2_+2.64	8.4205	0.69
− 2+1	y_1_=− 0.16x_1_+1.00	3.8425	0.57	y_2_=3.20x_2_+2.14	7.1975	0.67
− 1+0.5	y_1_=− 0.07x_1_+0.87	3.9316	0.17	y_2_=3.18x_2_+1.95	7.1807	0.65
− 0.5+0.25	y_1_=0.25x_1_+0.66	4.2528	0.54	y_2_=2.77x_2_+1.47	6.7736	0.54
− 0.25+0.15	y_1_=0.14x_1_+0.66	4.1409	0.45	y_2_=3.05x_2_+1.60	7.0506	0.55
− 0.15+0.075	y_1_=0.29x_1_+0.99	4.2891	0.88	y_2_=2.01x_2_+1.52	6.0092	0.59
− 0.075+0.045	y_1_=− 0.16x_1_+1.06	3.8354	0.66	y_2_=2.67x_2_+2.04	6.6743	0.66
− 0.045+0.038	y_1_=− 0.15x_1_+1.04	3.8521	0.59	y_2_=2.71x_2_+2.01	6.7104	0.67
− 0.038+0.025	y_1_=− 0.13x_1_+1.07	3.8681	0.60	y_2_=3.97x_2_+2.33	7.9703	0.70
− 0.025	y_1_=− 0.25x_1_+1.12	3.7507	0.79	y_2_=3.75x_2_+2.38	7.7449	0.71

Moreover, Fig. 7 shows the results of Menger model fitting for the fractal features of the macroporous structure of CSDs at different PSSs. Table 3 shows the linear fitting equations, fractal dimensions (*D*_M1_ and *D*_M1_) and *R*^2^ values for the pore channels of CSDs with pore diameters larger than 50 nm. As shown in Table 3, the *D*_M_ increased with increasing particle sizes, and the correlation coefficients for the Menger model were 0.16 < *R*^2^ < 0.89, indicating poor reliability for the fractal features of the macroporous structure determined with the Menger model. Larger *D*_M_ values indicate greater spatial geometric complexity of the macroporous channel shapes, and larger deviations from a smooth surface. *D*_M1_ increased and then decreased with decreasing particle sizes, and the *D*_M1_ of − 0.15+0.075 mm had the largest value (4.2891), which indicated that the macroporous structure of − 0.15+0.075 mm was more complex than those of the other PSSs. *D*_M2_ decreased and then increased with decreasing particle sizes, and the *D*_M2_ reached the lowest value (6.0092) at − 0.15+0.075 mm. *D*_M1_=3.7507 and *D*_M2_=7.7449 at − 0.025 mm were smaller than those at − 2 mm, indicating that the macroporous pore structures of the CSDs with the coarse PSS were more complicated, while the number of macropores in the fine PSS was less complicated. This was attributed to the presence of structures such as cracks, concave and convex structures, and steps in the coarse PSS of the CSD, which was consistent with the *SSA* test results in Table 2 and Fig. 5.

### Fractal characterization of the image processing method

**Figure 8:**
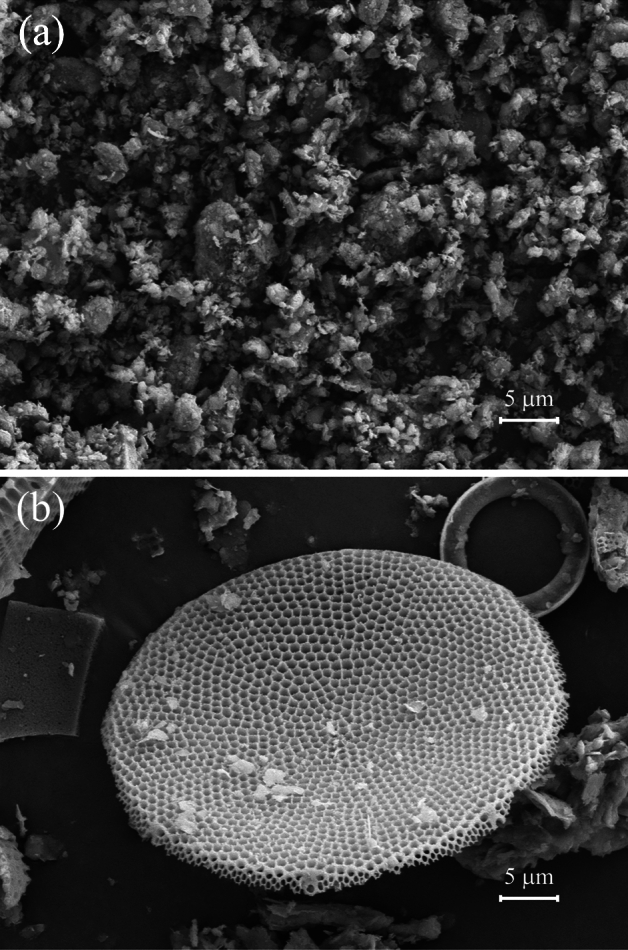
SEM images of CSD for − 2 mm (**a**) and − 0.025 mm (**b**).

Fig. 8 shows SEM images of the − 2 mm and − 0.025 mm samples. As shown in Fig. 8a, the mineral composition in the CSD was complex, with a high content of gangue minerals, diatomite surface covering and pore channel plugging with clay minerals, and poor pore structure characteristics. As shown in Fig. 8b, the diatoms in the fine PSS of the CSDs had complete morphologies, more 3D pore channels, and better pore structure characteristics. In comparison, the diatoms are mainly enriched in the fine PSSs of the CSDs and showed better pore structure characteristics.

**Figure 9:**
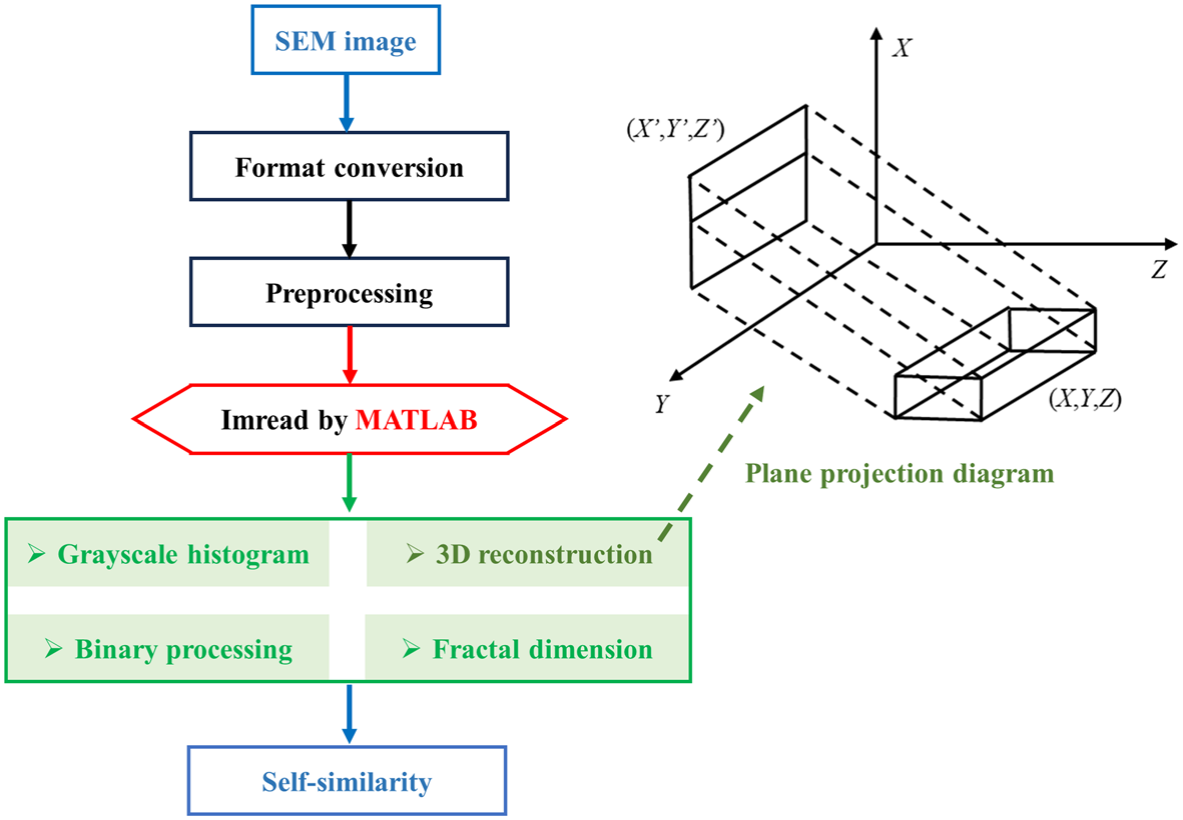
SEM image processing and analyses (grayscale histogram, binary processing, 3D reconstruction and fractal dimension) were performed with MATLAB.

**Figure 10:**
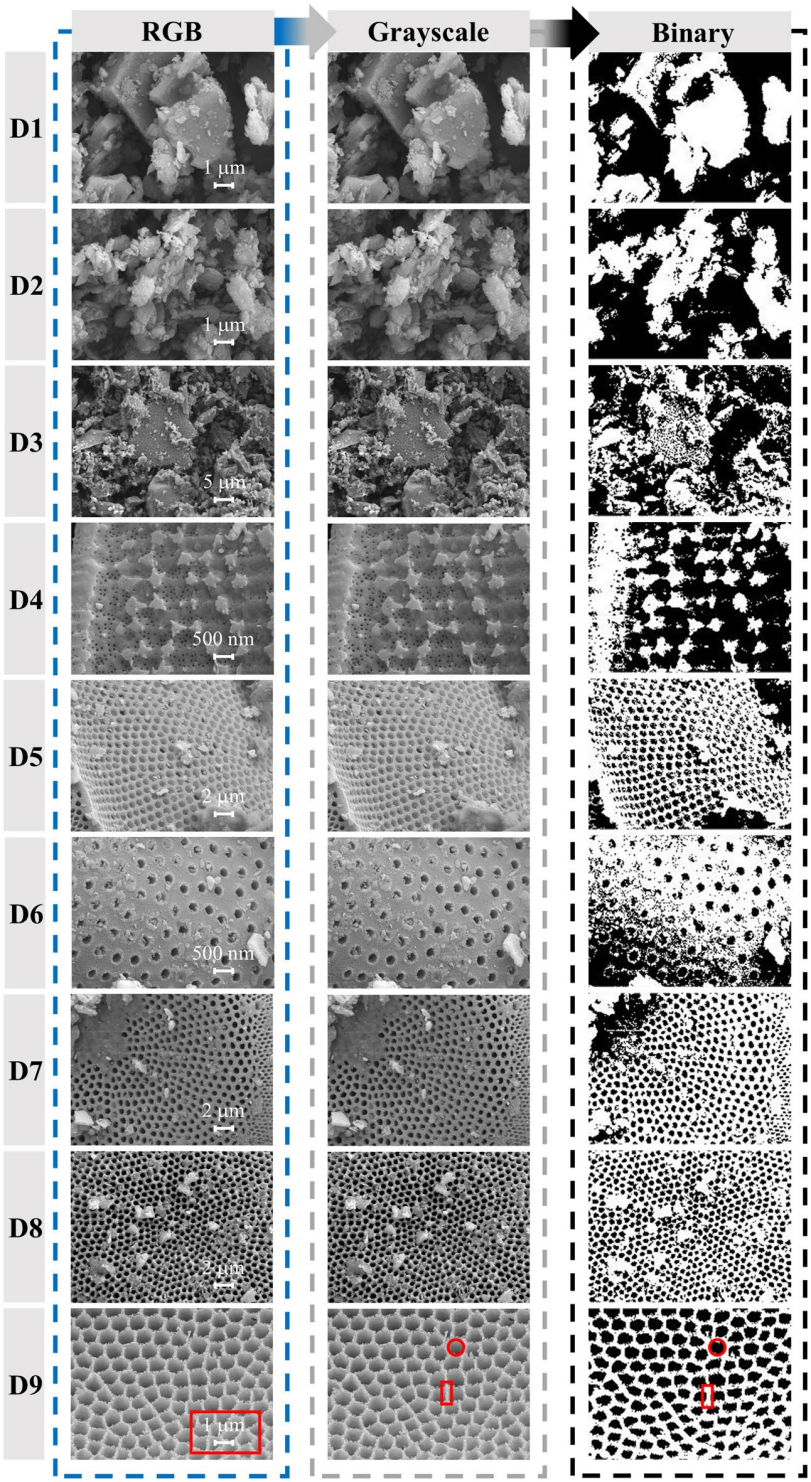
RGB, grayscale, and binary images (D1: − 2+1 mm, D2: − 1+0.5 mm, D3: − 0.5+0.25 mm, D4: − 0.25+0.15 mm, D5: − 0.15+0.075 mm, D6: − 0.075+0.045 mm, D7: − 0.045+0.038 mm, D8: − 0.038+0.025 mm, D9: − 0.025 mm).

SEM image analyses and self-similarity fractal characterizations of the CSDs with different PSSs were carried out via image processing (Fig. 9). As shown in Fig. 10, in the SEM images of the CSDs with different PSSs, the selected images had more obvious fractal features and thus were more representative. The black areas in the image were the pore channels, and the white areas were the particles and skeletons. As seen in the RGB images, the CSD was mainly composed of rounded diatom particles, which were structured and connected together by the outer rings. Due to this structural feature, there were many pore channels of different diameters between and within the diatom particles. As the particle sizes of the CSD decreased, the diatom surface was covered, and pore channels were blocked by fewer gangue minerals, exposing more pore channels. At − 0.025 mm, the macropores between the diatom particles gradually disappeared, and the internal mesopores were exposed. The grayscale thresholds of the CSDs with different PSSs were obtained from algorithms (6–7) as 106 (− 2+1 mm), 113 (− 1+0.5 mm), 101 (− 0.5+0.25 mm), 119 (− 0.25+0.15 mm), 151 (− 0.15+0.075 mm), 137 (− 0.075+0.045 mm), 100 (− 0.045+0.038 mm), 91 (− 0.038+0.025 mm), and 144 (− 0.025 mm). The grayscale images processed with the Eq. (8) algorithm are shown in binary images, with the background pixels as particles (white) and the target pixels as pores (black).

**Figure 11:**
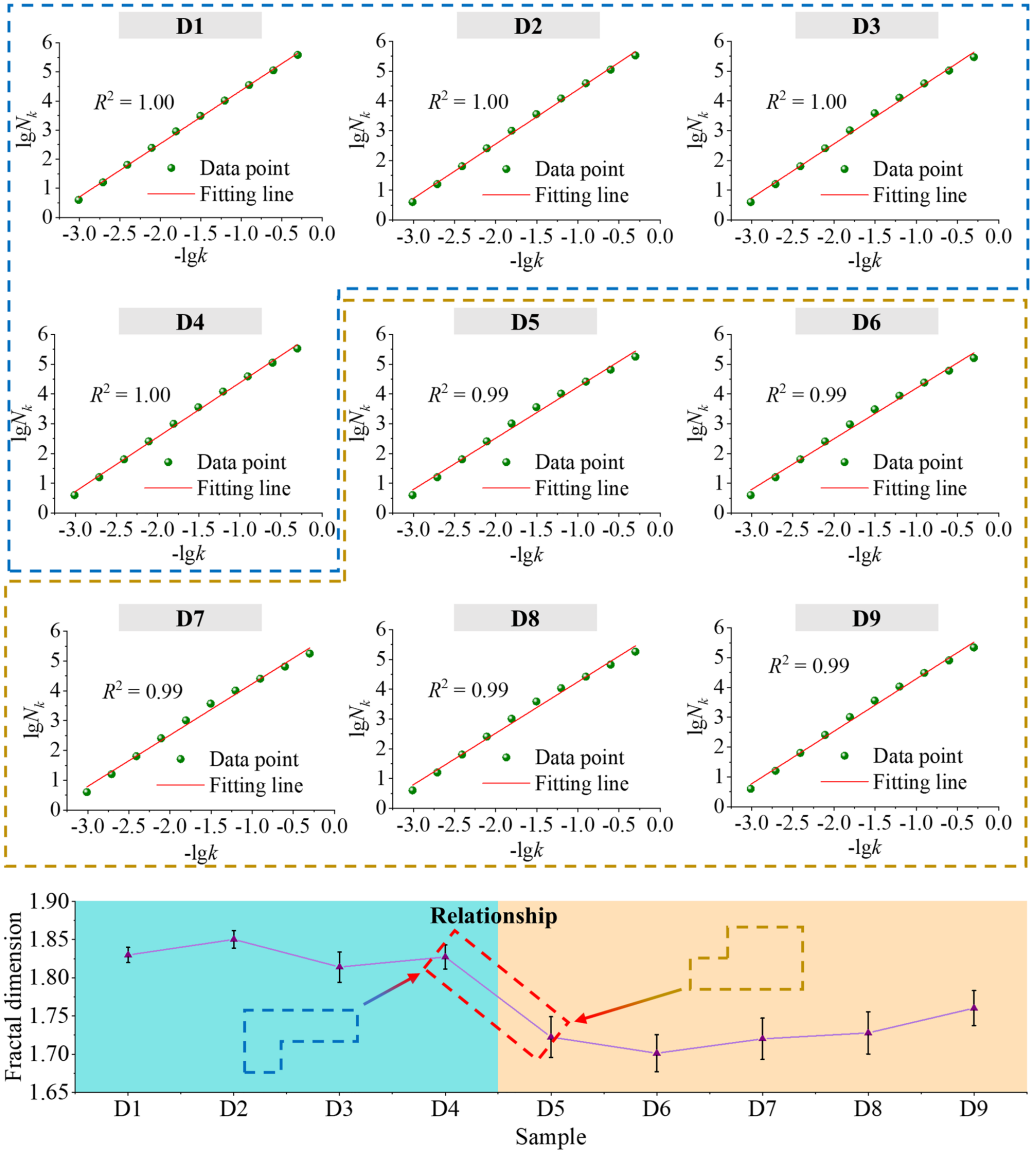
Linear fits of bi-logarithmic plots of fractal dimensions and PSSs (D1: − 2+1 mm, D2: − 1+0.5 mm, D3: − 0.5+0.25 mm, D4: − 0.25+0.15 mm, D5: − 0.15+0.075 mm, D6: − 0.075+0.045 mm, D7: − 0.045+0.038 mm, D8: − 0.038+0.025 mm, D9: − 0.025 mm).

According to Eq. (9), a plot of lg(1/*k*) vs. lg*N*_*k*_ was subject to least squares fitting in the double logarithmic coordinate plane, and the slope of the resulting straight line was the number of box dimensions. The binary images were imported into the written MATLAB program, and the fitting results are shown in Fig. 11. The *N*_*k*_ values of the CSDs with different PSSs are shown in Table 4.

**Table 4:** *N*_k_ for different PSSs of the CSDs.

Size/mm	*N*_*k*_ for different *k*_*n*_									
	2	4	8	16	32	64	128	256	512	1024
− 2+1	383135	113203	35255	10364	3079	903	247	64	16	4
− 1+0.5	418080	125177	39520	11678	3427	979	256	64	16	4
− 0.5+0.25	295480	104638	38754	12799	3824	1021	256	64	16	4
− 0.25+0.15	337815	112170	39090	12059	3583	999	256	64	16	4
− 0.15+0.075	179347	65574	25970	10247	3626	1021	256	64	16	4
− 0.075+0.045	161221	60756	24166	8772	3049	959	255	64	16	4
− 0.045+0.038	176966	65035	25473	10221	3716	1022	256	64	16	4
− 0.038+0.025	182751	66835	26423	10883	3860	1022	256	64	16	4
− 0.025	220307	81121	30778	10733	3632	1020	256	64	16	4

As shown in Fig. 11, the SEM images of the CSDs with different PSSs had better fractal characteristics after binarization, and the correlation between the experimental data points and the fitted straight line was high. Thus, the microstructural changes of the CSDs with different PSSs were described by the fractal box dimension. Combined with the SEM images in Fig. 10, the changes in fractal features were divided into two stages: (1) from the perspective of D1 to D4, the variations in the box dimensions of the coarse PSS were not significant and relatively large due to the clogging and encapsulation of the mesopores with gangue minerals and the presence of structures such as cracks, concavity and convexity, and steps; (2) from the perspective of D5–D9, the box dimensions of the fine PSS increased with decreasing particle sizes, which was consistent with N_2_ isothermal adsorption–desorption, FHH modelling, and SEM imaging. However, the overall box dimensions of D5–D9 were smaller than those of D1–D5. This is because the mineralogical characteristics of the CSD had a significant impact on the box dimension, while the effects of particle gaps and gangue mineral particles on the box dimension were relatively small for fine PSSs. Therefore, the fractal box dimension characterized the changes in the self-similar pore structure and the changes in the pore distribution after the disappearance of structural properties for CSDs at different PSSs discussed in this study.

**Figure 12:**
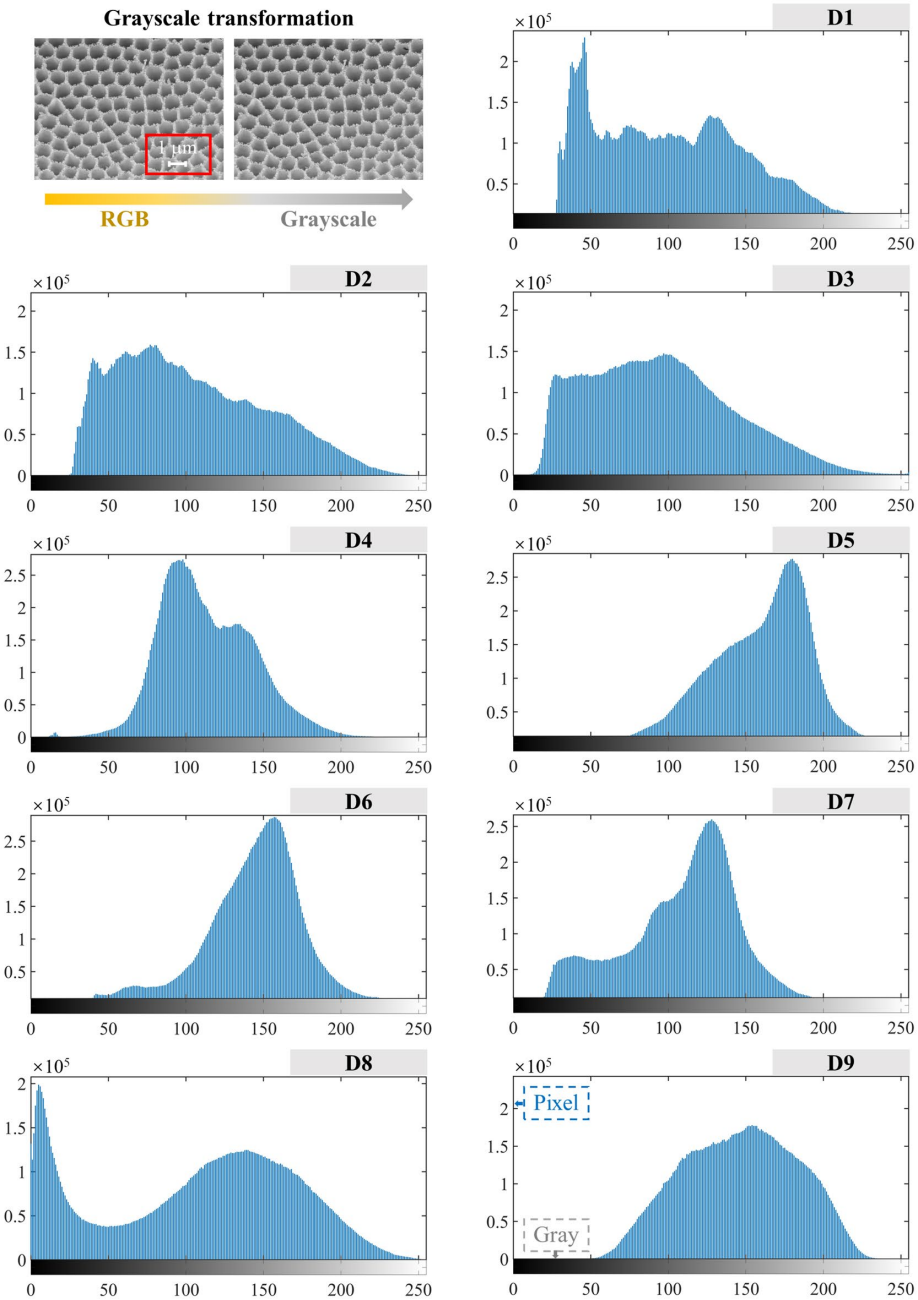
Grayscale histograms of SEM images with different PSSs (D1: − 2+1 mm, D2: − 1+0.5 mm, D3: − 0.5+0.25 mm, D4: − 0.25+0.15 mm, D5: − 0.15+0.075 mm, D6: − 0.075+0.045 mm, D7: − 0.045+0.038 mm, D8: − 0.038+0.025 mm, D9: − 0.025 mm).

**Figure 13:**
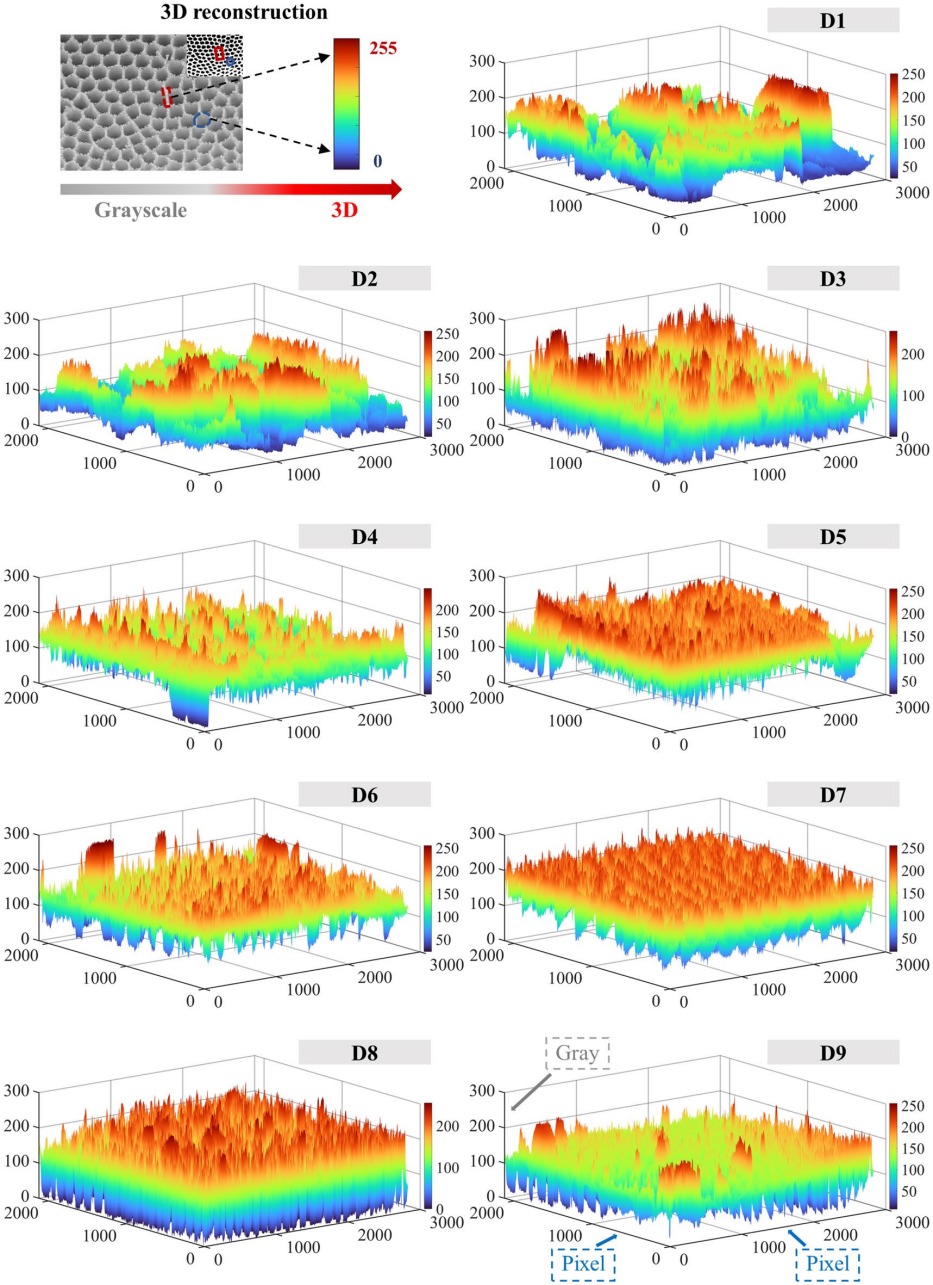
3D reconstructions of CSDs with different PSSs (D1: − 2+1 mm, D2: − 1+0.5 mm, D3: − 0.5+0.25 mm, D4: − 0.25+0.15 mm, D5: − 0.15+0.075 mm, D6: − 0.075+0.045 mm, D7: − 0.045+0.038 mm, D8: − 0.038+0.025 mm, D9: − 0.025 mm).

To visualize the self-similarity 3D pore structures of the CSD, 3D SEM images of the different PSSs were reconstructed. (1) Based on the SEM grayscale image, the grayscale histogram was created by the imhist function in MATLAB software (Fig. 12). The horizontal axis of the histogram represents the grayscale value, the vertical axis represents the pixel, and each vertical line represents the number of pixels contained under the grayscale value, i.e., the frequency of each grayscale value appearing in the whole SEM image of the CSD. The grayscale values ranged from 0 to 255, and the blacker the image was, the smaller the grayscale value, and the brighter the image was, the larger the grayscale value; i.e., the pixels with grayscale values of 0 were black and represented apertures in the SEM image, the pixels with grayscale values of 255 were white and represented particles, and the other pixels were between black and white. (2) The preprocessed image was added to the search path of the MATLAB software, and then the image information was extracted by the imread function. The pixels of the CSD image used in this experiment were 2837 × 2121, the pixels of the image were used as the length and width, the grayscale value was taken as the height to obtain the 3D dataset, and the data were stored in a CAT file for subsequent use. (3) The acquired 3D data were projected on the apparent plane with the body projection method, i.e., the acquired 3D body data of the SEM image were projected onto the plane that was observed visually according to the projection method, and then the projected equivalent surface was drawn with the isosurface function. (4) The colour, shadow and display effect of the image were set to make the 3D model more intuitive, and comprehensive 3D reconstructions of the CSDs were achieved at different PSSs. The final 3D reconstruction is shown in Fig. 13. The reconstructed 3D image provided a good 3D display, the undulations of the self-similarity structures of the CSDs were clearly observed, and the sizes, shapes and distributions of the particles and pores were all consistent with the actual pore structure characteristics of CSDs at different PSSs.

## Conclusions

CSD comprises coal-series waste through open piling and backfilling of mining areas, which causes a serious waste of resources and great pressure on the environment. In this study, we took CSD as the research object and PSS as the entry point and conducted a self-similarity study based on an analytical gas adsorption method, an image segmentation method, an optimal threshold method and a 3D reconstruction method to illustrate the self-similarity 3D pore structures of CSDs at different PSSs.

The main chemical component of the CSD was SiO_2_ (59.22%), and the LOI was 10.50%; the main mineral component was opal, with a content of approximately 12.39%, which accounted for approximately one-eighth of the total mineral content. Opal was the main constituent mineral of the diatom skeleton, with a chemical composition of SiO_2_·nH_2_O, and formation of the pore structure (diatom skeleton) was attributed to the 3D constituent unit built by SiO_4_ tetrahedra. The self-similarity pore structure was poorly characterized due to the low SiO_2_ content and the complexity of the associated minerals in the CSD.

The pore structures of the CSDs with different PSSs were dominated by mesopores and macropores, with basically no micropores. The *SSA* and *V*_mes_ values were positively correlated with the amount of N_2_ adsorbed and negatively correlated with the PSS in general. The fractal dimensions calculated with the FHH model were 2.51/2.68 for − 0.025 mm and 2.48/2.64 for − 2 mm, suggesting that the mesopore structure of fine PSS was complex, whereas macropores were predominantly present in the coarse PSS. This was attributed to clogging and encapsulation of gangue minerals as well as the presence of structures such as cracks, concave and convex cracks, and steps that gave the CSD different self-similarity pore structural characteristics at different PSSs.

The grayscale thresholds, binarized images, grayscale histograms, 3D reconstruction images, and a series of box dimensions calculated from the SEM images of CSD at different PSSs in the image processing method enabled intuitive indications of the microstructure of the CSDs as well as self-similarity features. Meanwhile, the reconstructed 3D image provided a good 3D display, the undulations of the self-similarity functional structures of the CSDs were clearly observed, and the sizes, shapes, and distributions of the particles and pore channels matched the pore structures of the actual CSDs. Self-similarity studies based on particle sizes are important for the functional application of CSDs.

### Supplementary Information

Supplementary Information.

## Data Availability

All data generated or analysed during this study are included in this published article [and its supplementary information files].

## References

[1] Guo J, Zhao Y, Cai L, Zhang F, Bai X, Wang W (2023). Exact self-similar patterns of atomic-layer boron nitride with Koch fractal complexity. ACS Mater. Lett..

[2] Wang J, Wang X, Ding S, Ashour A, Yu F, Lv X, Han B (2023). Micro-nano scale pore structure and fractal dimension of ultra-high performance cementitious composites modified with nanofillers. Cem. Concr. Compos..

[3] Mandelbrot B (1967). How long is the coast of Britain?. Stat. Self-Similar. Fract. Dimens. Sci..

[4] Man J, Gao W, Yan S, Liu G, Hao H (2017). Preparation of porous brick from diatomite and sugar filter mud at lower temperature. Constr. Build. Mater..

[5] Guo Y, Huang Y, Li J, Ouyang S, Fan B, Liu Y, Hou G (2023). Preparation of the geopolymer grouting material by coal-based solid wastes for the aquiclude key strata and its application. Constr. Build. Mater..

[6] Guo Z, Liu J, Guo Z, Fan R, Zhou E (2022). Research progress on comprehensive utilization of associated mineral resources in coal-bearing strata in China. Conserv. Util. Miner. Resour..

[7] Cao D, Qin G, Zhang Y, Ning S, Wu G, Chen M, Qin Y, Zhu S, Zhu H (2016). Classification and combination relationship of mineral resources in coal measures. J. China Coal Soc..

[8] Huang B, Zhao X, Yu B, He G, Yue Z, Yang C, Wang C, Meng Q, Yang Y, Liu J, Feng X, Chen D, Xing Y, Zhu W, Duan X, Ju J (2022). Research framework of theory and technology for coordinated mining of coal and its co-existed and associated strategic metal minerals. J. China Coal Soc..

[9] Pugliese L, Canga E, Hansen HCB, Kjærgaard C, Heckrath GJ, Poulsen TG (2023). Long-term phosphorus removal by Ca and Fe-rich drainage filter materials under variable flow and inlet concentrations. Water Res..

[10] Xuan Y, Gao H, Tian H, Hu Z, Ma J, Yu Q (2023). Enhancement effect of Ce addition on Mn_3_O_4_/diatomite sorbent for moderate temperature flue gas desulfurization. Chem. Eng. J..

[11] Dong X, Chen Z, Tang A, Dionysiou DD, Yang H (2022). Mineral modulated single atom catalyst for effective water treatment. Adv. Funct. Mater..

[12] Nikolaev, V. K., Skvortsov, A. A., Lukyanov, M. N., Skvortsov, P. A., Gridasova, E. A. & Dmitrevsky, A. A. Relationship of dynamic and static modules of elasticity of ceramics based on diatomite. *Ceram. Int.* (2023).

[13] Sriram G, Kigga M, Uthappa UT, Rego RM, Thendral V, Kumeria T, Jung H, Kurkuri MD (2020). Naturally available diatomite and their surface modification for the removal of hazardous dye and metal ions: A review. Adv. Colloid Interfac..

[14] Ren M, Chen T, Gao X, Su A (2023). Long term properties of cement-based material incorporated with n-octadecane/diatomite composite SSPCM. Cem. Concr. Compos..

[15] Wen X, Feng W, Li X, Yang J, Du R, Wang P, Li H, Song L, Wang Y, Cheng M, He J, Shi J (2023). Diatomite-templated synthesis of single-atom cobalt-doped MoS_2_/carbon composites to boost sodium storage. Adv. Mater..

[16] Zhou J, Cheng L, Ma Z, Weng X, Gao J (2023). Integrated nanostructures of TiO_2_/g-C_3_N_4_/diatomite based on low-grade diatomite as efficient catalyst for photocatalytic degradation of methylene blue: performance and mechanism. Catalysts..

[17] Weller T, Sann J, Marschall R (2016). Pore structure controlling the activity of mesoporous crystalline CsTaWO_6_ for photocatalytic hydrogen generation. Adv. Energy Mater..

[18] Saleem A, Zhang Y, Usman M, Haris M, Li P (2022). Tailored architectures of mesoporous carbon nanostructures: From synthesis to applications. Nano Today.

[19] Wang J, Cheng C, Zheng X, Idrobo JC, Lu AY, Park JH, Shin BG, Jung SJ, Zhang T, Wang H, Gao G, Shin B, Jin X, Ju L, Han Y, Li LJ, Karnik R, Kong J (2023). Cascaded compression of size distribution of nanopores in monolayer graphene. Nature.

[20] Schouwenaars R, Jacobo VH, Ortiz A (2017). The effect of vertical scaling on the estimation of the fractal dimension of randomly rough surfaces. Appl. Surf. Sci..

[21] Sun Z, Mao J, Hu Z, Zheng S (2017). Study on pilot-scale centrifugal separator for low-grade diatomite purification using response surface methodology. Particul. Sci. Technol..

[22] Chen F, Miao Y, Ma L, Zhan F, Wang W, Chen N, Xie Q (2020). Optimization of pore structure of a clayey diatomite. Particul. Sci. Technol..

[23] Wakeel MIA (2009). Characterization and process development of the Nile diatomaceous sediment. Int. J. Miner. Process..

[24] Li X, Xu W, Yang Y, Li B, Pan G, Chen N, Xie Q (2023). Optimization of diatom-based blotting materials and their efficient selective adsorption of Pb(II). Mater. Today Commun..

[25] Xu P, Wei R, Wang P, Li X, Yang C, Shen T, Zheng T, Zhang G (2023). CuFe_2_O_4_/diatomite actuates peroxymonosulfate activation process: Mechanism for active species transformation and pesticide degradation. Water Res..

[26] Ren Z, He Y, Zheng R, Guo Z, Gao H, He X, Wu F, Ji X (2022). The preparation and characterization of calcined diatomite with high adsorption properties by CaO hydrothermal activation. Colloid. Surface. A.

[27] Xie D, Qi J, Zhang Z (2000). A constitutive laws considering soil structural properties. China Civ. Eng. J..

[28] Hu Z, Zheng S, Sun Z, Chen Y, Yan Y (2017). Influence of pore structure on humidity control performance of diatomite. Sci. Technol. Built En..

[29] Jiang F, Zhang L, Jiang Z, Li C, Cang D, Liu X, Xuan Y, Ding Y (2019). Diatomite-based porous ceramics with high apparent porosity: Pore structure modification using calcium carbonate. Ceram. Int..

[30] Smith MA, Lobo RF (2010). A fractal description of pore structure in block-copolymer templated mesoporous silicates. Micropor. Mesopor. Mat..

[31] Wang J (2011). Hunningtu ditan jishu yu gaoxingneng hunningtu.

[32] Wang Y, Ji F, Gu H, Ding J (2017). Fractal characteristics of natural sedimentary diatomaceous earth based on SEM images. Hydro. Sci. Eng..

[33] Niu F, Zhang H, Zhang J (2021). Three-dimensional reconstruction of SEM image of hematite flocs by MATLAB software. China Min. Mag..

[34] Zhang X, Wang C, Ma D (2012). 3D visualization and fractal dimension of soft clay's microstructure surface undulation. J. Basic Sci. Eng..

[35] Wang Z, Zhang QP, Guo F, Ma H, Liang ZH, Yi CH, Zhang C, Chen CF (2024). Self-similar chiral organic molecular cages. Nat. Commun..

[36] Fu J, Hui T, Gao M, Xu D, Zhou C, Qiu M (2024). Magic self-similar pattern of fractal materials: Synthesis, properties and applications. Coordin. Chem. Rev..

[37] JC/T 414-2017, Diatomite. Ministry of Industry and Information. Technology of the People’s Republic of China, Beijing, 2017, pp 1–14

[38] Liu Q, Li M, Wang S, Lv S, Han F, Xi Y, Cao Z, Ouyang J (2023). Ultrathin 3D CoMn nanoflowers coupled diatomite for highly efficient catalytic oxidation of CO and propane. Chem. Eng. J..

[39] Liu Z, Zhang Z, Zhang S, Zhang Y, Yuan Z, Li H, Jiang J (2022). Microstructure and performance characterization of thermal-insulating and water-resistive aerogel incorporated cement-based materials. Cem. Concr. Compos..

[40] Łach M, Pławecka K, Marczyk J, Ziejewska C, Krupa MH, Nykiel M, Hebda M, Miernik K, Mierzwiński D, Korniejenko K, Mikuła J, Smoroń K (2023). Use of diatomite from Polish fields in sustainable development as a sorbent for petroleum substances. J. Clean. Prod..

[41] Wei W, Chen Z, Hao D, Liu X, Ni BJ (2021). Natural diatomite mediated continuous anaerobic sludge digestion: Performance, modelling and mechanisms. J. Clean. Prod..

[42] Hu Z, Zheng S, Jia M, Dong X, Sun Z (2017). Preparation and characterization of novel diatomite/ground calcium carbonate composite humidity control material. Adv. Powder. Technol..

[43] Hu Z, Zheng S, Tan Y, Jia M (2017). Preparation and characterization of diatomite/silica composite humidity control material by partial alkali dissolution. Mater. Lett..

[44] Zhu Y, Liu H, Wang T, Wang Y, Liu H (2023). Evolution of pore structures and fractal characteristics of coal-based activated carbon in steam activation based on nitrogen adsorption method. Powder Technol..

[45] Han X, Feng J, Wang B (2023). Relationship between fractal feature and compressive strength of fly ash-cement composite cementitious materials. Cem. Concr. Compos..

[46] Olmez Y, Koca GO, Tanyildizi E, Sengur A (2023). Multilevel image thresholding based on Renyi’s entropy and golden sinus algorithm II. Neural Comput. Appl..

[47] Qiao L, Liu K, Xue Y, Tang W, Salehnia T (2024). A multi-level thresholding image segmentation method using hybrid Arithmetic Optimization and Harris Hawks Optimizer algorithms. Expert Syst. Appl..

[48] Yao K, Li K, Wang Z, He M (2023). The Hausdorff dimension of Hadamard fractional integral of a fractal function. Chaos Soliton. Fract..

[49] Wang X, Zhao C, Yuan X (2022). A review of fractal functions and applications. Fractals.

[50] Moriguchi K (2023). Estimation of fractal dimension of trees using LiDAR point data with sequential data decimation. Remote Sens. Environ..

[51] Chen F, Muhammad K, Wang SH (2020). Three-dimensional reconstruction of CT image features based on multi-threaded deep learning calculation. Pattern Recogn. Lett..

[52] Yi J, Li L, Wang L, Xue F (2017). Calculation method of 3D porosity and fractal dimension of solidified sludge’s microstructure by GIS. J. Cent. S. U. Sci. Technol..

[53] Wu, Z., Wang, L., Li, Y., Dai, S. & Zhang, D. Head CT image segmentation and three-dimensional reconstruction technology based on human anatomy. *Comput. Intel. Neurosc.* 7091476 (2022). 10.1155/2022/7091476 PMC9225843 35755748

[54] Niu F, Zhang H, Zhang J (2021). Three-dimensional reconstruction of SEM image of hematite flocs by MATLAB software. China Min. Mag..

[55] Jiang Y, Wang Y, Wang S (2011). 3D reconstruction for CT images of expansive soil based on MATLAB. J. Southwest U. (Nat. Sci. Ed.).

[56] Sun M, Zou C, Xin D (2020). Pore structure evolution mechanism of cement mortar containing diatomite subjected to freeze-thaw cycles by multifractal analysis. Cem. Concr. Compos..

[57] Wei PH (1935). Zeitschrift fuer Kristallographie. Kristallgeometrie, Kristallphysik, Kristallchemie, Nabu Press.

